# Attitudes of Anesthesiologists toward Artificial Intelligence in Anesthesia: A Multicenter, Mixed Qualitative–Quantitative Study

**DOI:** 10.3390/jcm12062096

**Published:** 2023-03-07

**Authors:** David Henckert, Amos Malorgio, Giovanna Schweiger, Florian J. Raimann, Florian Piekarski, Kai Zacharowski, Sebastian Hottenrott, Patrick Meybohm, David W. Tscholl, Donat R. Spahn, Tadzio R. Roche

**Affiliations:** 1 Institute of Anaesthesiology, University and University Hospital of Zurich, 8091 Zurich, Switzerland; 2 Department of Anaesthesiology, Intensive Care and Pain Medicine, Frankfurt University Hospital, 60590 Frankfurt am Main, Germany; 3 Department of Anaesthesiology, Intensive Care, Emergency and Pain Medicine, University Hospital Wuerzburg, 97080 Wuerzburg, Germany

**Keywords:** artificial intelligence, machine learning, anesthesia, anesthesiology, qualitative research, clinical decision support

## Abstract

Artificial intelligence (AI) is predicted to play an increasingly important role in perioperative medicine in the very near future. However, little is known about what anesthesiologists know and think about AI in this context. This is important because the successful introduction of new technologies depends on the understanding and cooperation of end users. We sought to investigate how much anesthesiologists know about AI and what they think about the introduction of AI-based technologies into the clinical setting. In order to better understand what anesthesiologists think of AI, we recruited 21 anesthesiologists from 2 university hospitals for face-to-face structured interviews. The interview transcripts were subdivided sentence-by-sentence into discrete statements, and statements were then grouped into key themes. Subsequently, a survey of closed questions based on these themes was sent to 70 anesthesiologists from 3 university hospitals for rating. In the interviews, the base level of knowledge of AI was good at 86 of 90 statements (96%), although awareness of the potential applications of AI in anesthesia was poor at only 7 of 42 statements (17%). Regarding the implementation of AI in anesthesia, statements were split roughly evenly between pros (46 of 105, 44%) and cons (59 of 105, 56%). Interviewees considered that AI could usefully be used in diverse tasks such as risk stratification, the prediction of vital sign changes, or as a treatment guide. The validity of these themes was probed in a follow-up survey of 70 anesthesiologists with a response rate of 70%, which confirmed an overall positive view of AI in this group. Anesthesiologists hold a range of opinions, both positive and negative, regarding the application of AI in their field of work. Survey-based studies do not always uncover the full breadth of nuance of opinion amongst clinicians. Engagement with specific concerns, both technical and ethical, will prove important as this technology moves from research to the clinic.

## 1. Introduction

The artificial intelligence (AI) revolution in medicine is well underway [1]. The list of potential applications of AI and its subfield machine learning (ML, here used as synonyms for technologies that aim to replicate human cognitive functions with computer algorithms) is ever-expanding, and many research programs are now bearing clinical fruit [2,3]. Following the first FDA approval of an AI algorithm in 2016, barely a year later, 64 AI-based technologies had been brought to market [1]. At the same time, although the literature is in its infancy, these technologies appear to be starting to perform as well as human physicians [4,5]. Thus, it seems likely that the roles AI-based technologies play in medicine will continue to grow.

In the field of anesthesia, researchers have thus far applied AI to the depth of anesthesia monitoring, event and risk prediction, ultrasound guidance, and even operating room management [6]. While the holy grail of closed-loop, autonomous anesthesia control systems remains some ways off [7], new, commercially available blood pressure control systems are already bringing AI technology into the operating theater [8]. All this suggests that, in the foreseeable future, a wide range of anesthesia providers will need to become familiar with AI-based technologies and integrate them into their practices.

Little is known, however, as to what anesthesia providers think about this technological development. To date, there have been no comprehensive qualitative studies of attitudes toward and/or knowledge of AI in anesthesia. Existing, related work from other specialties has established that computer scientists rate the potential benefits of AI as higher—and potential for patient harm lower—than clinicians [9] and that the more familiar clinicians are with AI, the more likely they are to hold positive views about the technology [10,11,12]. However, it is unclear whether the same holds true for anesthesia specifically. Moreover, the existing data are largely survey-based, and, therefore, limited in their ability to capture the same breadth and nuance as more open-ended qualitative work [13,14].

The implementation of any technology depends only in part on technological advantage. Other factors, including but not limited to “blind spots” or weaknesses inherent to the technology itself [15], as well as ethics, regulatory hurdles, and the attitudes and opinions the end users hold, also play a fundamental role [16]. Indeed, many technologies that are clinically useful do not find widespread acceptance, and vice versa [17]. Success in integrating AI into the clinic will instead depend on collaborating with anesthesiologists, respecting their deep knowledge of clinical anesthesia, and seeking first to understand their attitudes and opinions regarding AI [18].

We considered that more should be known about how much anesthesiologists already know and what they think about AI. To best explore this research gap, we chose a qualitative study design. These opinions were collected in individual interviews and an accompanying survey.

## 2. Materials and Methods

This study used thematic analysis, within an overall constructivist framework, to explore anesthesiologists’ perceptions of AI. By discussing and challenging established assumptions, the study authors sought to maintain reflexivity in the collection, data analysis, and writing processes. As guides for carrying out high-quality qualitative studies, the SRQR guidelines and COREQ checklist served as references throughout this process [19,20].

### 2.1. Study Design

This was an investigator-initiated, prospective, mixed qualitative–quantitative study conducted in two parts. The first, qualitative part of the study consisted of face-to-face, structured interviews with physician anesthesiologists (*n* = 21). These interviews were analyzed for key themes, and, from these themes, six representative statements were derived. Subsequently, in the second part of the study, these representative statements were sent as a questionnaire with five-point Likert-type, scalable answers to study participants (*n* = 70) for rating.

### 2.2. Study Setting

The study was carried out in the university hospitals of the cities of Zurich, Switzerland (Universitätsspital Zürich, USZ), Würzburg, Germany (Universitätsklinikum Würzburg, UKW), and Frankfurt, Germany (Universitätsklinikum Frankfurt, UKF). All three centers are university-affiliated, tertiary-referral teaching hospitals offering the full range of anesthetic subspecialties. The questionnaire for part two of the study was sent via email to participating anesthesiologists at all three centers.

### 2.3. Study Participants

The study participants were all practicing physician anesthesiologists. For part one of the study, we recruited participants from the study centers in Würzburg and Frankfurt by availability on the days of data collection. After 21 interviews, theme saturation was reached [21]. In the second part of the study, a survey was sent to physician anesthesiologists across all three study sites (to a sample including the original 21 interviewees). Of 70 invites, 49 responses were received, leading to a response rate of 70%.

### 2.4. Part One: In-Depth Interviews

In part one of the study, structured interviews were used to explore study participants’ thoughts and opinions on AI and ML in anesthesia. Informed by the existing literature on AI in medicine [3,6,22], including previous, related qualitative work [9,10,11,12,23,24,25,26], author DH designed an interview comprising five open-ended questions. In discussion with TRR, these were then revised to form the final questionnaire (see Appendix A). Anticipating that study participants might not be familiar with either the concepts or examples of AI or ML in anesthesia, prompts were designed (including recent research articles) to be shown to interviewees after questions one and two to aid further discussion.

Study authors DH, GS, and AM then conducted face-to-face, structured interviews with the study participants. Interviews were carried out at UKW and UKF in a quiet office environment removed from the clinical areas of the hospital. There was no time limit, but interviews tended to last approximately 10 min. They were recorded using an iPhone (Apple Inc., Cupertino, CA, USA) and later transcribed verbatim using Trint (Trint Limited, London, UK). The transcripts were then manually checked for accuracy and completeness. These were then translated into English using the neural machine translation service DeepL (DeepL SE, Cologne, Germany) before being manually checked for accuracy.

The interviews were parsed into discrete statements, and these statements were coded using an inductive approach to thematic analysis [27,28]. Study author DH read and provisionally coded (i.e., applied descriptive labels to) statements from the first 15 transcripts. Coded statements were collated into first-order subthemes and, where informative, second-order subthemes. Independently, TRR reviewed the statements and formulated his own provisional coding schemes. The two reviewers then met to discuss and revise coding decisions, resulting in a set of definitive coding schemes. Next, all the statements were recoded separately by DH and TRR according to these finalized coding schemes (see Table A1, Appendix B). Finally, DH and TRR met to discuss any discrepancies in their coding and to agree on united coding for each statement.

### 2.5. Part Two: Online Survey

To construct the online survey, first, the most frequently recurring themes from the coded interviews in the first part of the study were used to create six representative statements. Then, each statement was reviewed for content and construct (content validity) by two members of the research group who were not involved in creating the statements but who have experience in survey creation and AI. Finally, two anesthesiologists from the University Hospital Zurich checked the six statements for comprehensibility (face validity).

The final six representative statements (see Appendix C) were answerable on a five-point Likert scale with the divisions “1, strongly disagree”, “2, disagree”, “3, neutral”, “4, agree”, and “5, strongly agree”. In order to quantify the level of agreement or disagreement with these statements in as wide a pool of practicing anesthesiologists as possible, a link to these statements in questionnaire format (Google Forms, Google LLC, Mountain View, CA, USA) was sent via email to all participants of a concurrently running anesthesia simulation study (and which included the original 21 interview candidates). The questionnaire remained active for a period of three weeks from July to August 2022. A single reminder email was sent halfway through this period.

### 2.6. Statistical Analysis

Data from part one of the study are reported as the number and percentage of responses corresponding to each code. The consistency of coding according to the final coding scheme between study authors DH and TR was assessed by calculating percent agreement and interrater reliability with Cohen’s kappa [29].

The results of the online survey are presented as numbers, medians, and interquartile ranges (IQR). The Wilcoxon signed-rank test was used as a test of statistical significance. We considered a deviation from neutral (i.e., a value of 3, “neutral”) as of practical significance and a *p*-value of <0.05 as statistically significant.

We used Microsoft Word, Microsoft Excel (Microsoft Corporation, Redmond, WA, USA), and R version 4.2.0 (R Foundation for Statistical Computing, Vienna, Austria) to manage and analyze our data.

## 3. Results

In the first half of 2022, we recruited 21 anesthesiologists from 2 centers for the first part of the study, the interview. In the second part of the study, the online survey, 49 anesthesiologists from across the 3 study centers participated (further study and participant characteristics are listed in Table 1).

### 3.1. Part One: In-Depth Interviews

**Figure 1:**
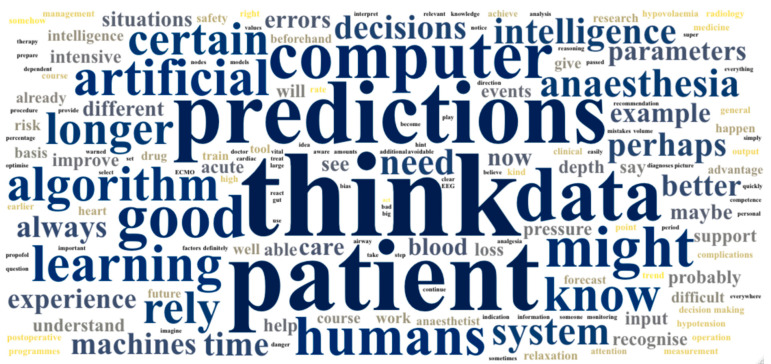
Graphical representation (R version 4.2.0) of the most common words in the participants’ collected answers to all questions. The word cloud makes more frequently used words appear larger.

In total, across all questions, 21 codes were derived via inductive coding by study authors DH and TRR. Interrater reliability, as measured by Cohen’s Kappa, was 0.908, and the percentage agreement was 91.4%. The 21 codes could be grouped into 3 main themes, namely, (1) a good pre-existing understanding of AI, (2) a balanced view of the pros and cons of AI as applied to anesthesia, and (3) a generally positive view of the use of AI to predict clinical events. An overview of responses corresponding to each question and code (as number and percentage, as well as example statements) organized by the themes above is provided in Table 2, Table 3 and Table 4. Figure 1 is a word cloud representing the most common words used by participants in their answers. Complete transcripts are available in Appendix D.

Statements derived from questions 1 and 2 demonstrated a good pre-existing understanding of AI. Participants’ statements mainly referenced AI as an “information technology” (44 of 90, 49%) and the “capabilities” (34 of 90, 38%) of AI. Of note, however, question 2 also revealed a lack of awareness of the applications of AI in anesthesia, with the majority of statements coded as “none” (23 of 42, 55%), followed by “research” (7 of 42, 17%) and “non-AI/-ML example” (7 of 42, 17%). Statements in response to question 3 demonstrated a balanced view of the pros (46 of 105, 44%) and cons (59 of 105, 56%) of AI as applied to anesthesia. Question 4 revealed a generally positive (32 of 67, 48%) or neutral (19 of 67, 28%) view of the use of AI to predict clinical events, with only a minority being negative in sentiment (6 of 67, 9%). Responses to question 5 were very varied, with statements referencing vital sign predictions (36 of 92, 39%), event type (19 of 92, 21%), treatment guide (17 of 92, 18%), and risk stratification (14 of 92, 15%) as potential targets for AI.

### 3.2. Part Two: Online Survey

**Figure 2:**
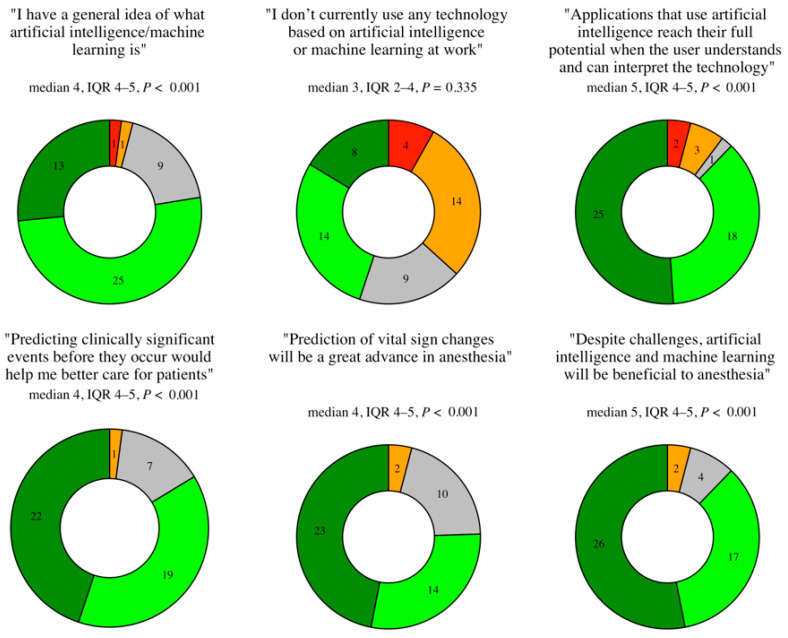
Part two survey results as donut diagrams of the number of responses per rating on a five-point Likert scale, and with medians, interquartile ranges, and *p*-values (strongly disagree = 1, disagree = 2, neutral = 3, agree = 4, strongly agree = 5). *n* = 49.

Overall participants were in agreement with the survey statements. A minimum of 37 of 49 participants (75%) agreed or strongly agreed with all statements, except for statement two, “I don’t currently use any technology based on AI or ML at work”, where there was a bimodal distribution of answers (14 of 49 participants (29%) agreed, and the same number disagreed with the statement). A more detailed breakdown of results is presented as donut diagrams in Figure 2.

## 4. Discussion

### 4.1. Principal Findings

The primary aim of this study was to explore what anesthesiologists already know and think about AI. We were able to derive three main themes from physician anesthesiologists’ responses to a series of in-depth interviews and a follow-up questionnaire, including (1) a good pre-existing understanding of AI, (2) a balanced view of the pros and cons of AI as applied to anesthesia, and (3) a generally positive view of the use of AI to predict clinical events.

Our dataset demonstrates a good level of pre-existing knowledge of AI in our sample of practicing anesthesiologists. Notably, all participants were able to give a definition of AI, with, at the interviews, only 4 of 90 statements (4%) coded as “little/no prior knowledge”. Furthermore, in the follow-up questionnaire, 38 of 49 survey participants (78%) “agreed” or “strongly agreed” that they had a general idea of what AI/ML is. In terms of content, many interviewees effectively paraphrased Arthur Samuel’s original definition of machine learning as “programming computers to learn from experience” [30]. Participant 3, for example, defined AI as “Computers [which] adjust and perfect their predictions based on experience”. Some participants were very well informed indeed. Participant 6, for example, offered a definition of a neural network: “Machine learning today [comprises] a neural network with different nodes, which have an input and an output, and the output can then be passed on to several nodes in the next level, and this output is weighted, i.e., amplified or degraded, before being passed on”.

In contrast, few interviewees were aware of the applications of AI in the field of anesthesia despite a fast-growing body of research literature. Other interviewees gave examples from unrelated specialties, especially radiology, or gave examples of a technology not based on AI principles. This knowledge gap was seen in part two of the study too, where there was a bimodal response (i.e., participants “agreed” and “disagreed” in equal measure) to the question “I don’t currently use any technology based on artificial intelligence or machine learning at work”.

What explains the gap between the ability to describe AI in the abstract and the inability to name a single application of AI in anesthesia, even if only from research? One explanation lies in the fact that, for the practicing clinician, AI is still largely an abstract technology and not yet in widespread clinical use (the Acumen Hypotension Prediction Index from Edwards Lifesciences remains the only FDA-approved example of an AI-based device [8]). Moreover, the average practicing physician consults the primary literature only relatively rarely [31]. Thus, there exists, as yet, no real clinical need to be familiar with AI-based technologies in the relatively little amount of time available to read research literature. Even in radiology, where AI has had the most impact, in one survey, a third of resident physicians had not read a single paper featuring AI in the preceding year [32]. Given this, it is perhaps unsurprising that concrete examples of AI implementations are relatively rare in our dataset.

Participants were generally able to take a balanced view of the application of AI to anesthesia. In this context, positive statements tended to reference more supposed technical capabilities of AI, for example, that an AI might be less prone to error, less biased, have more “experience” to draw on, or learn faster than a human operator might. Participant 8, for example, stated, “Machines don’t get tired, they don’t have bad days, they usually function better, they have a better memory than any human being and they have an unlimited capacity for learning”. In this regard, participants in this study echoed many of the putative benefits of AI, as described in the literature [2].

Negative statements also referenced technical issues, including biased training data or faulty or too rigid algorithms. This again mirrors much of the published literature on AI in medicine and also serves to underline the good pre-existing level of knowledge of AI in our cohort [33,34]. For example, “[It] is only as good as the material with which it has been trained” (participant 6); “In medicine … there is often a gender bias, especially in drug studies, and that is a challenge to overcome” (participant 18); or “The situation can be diverse or much more differentiated than an algorithm can reckon with” (participant 2).

However, in contrast to the positive statements, the majority of negative statements focused on human–computer interactions instead of technical factors. Here, participants focused on different challenges, for example, the potential for anesthesiologists to deskill, or become obsolete. For example, participant 11 stated, “The forward thinking you need as an anesthesiologist can be lost”. Similarly, and in common with previous survey data, in which concerns regarding obsolescence have also been reported, participant 20 stated, “We might make ourselves totally superfluous at some point” [9].

Another concern was the conflict that might arise were an AI to recommend a course of action that the human operator does not agree with. As other commentators have pointed out, an algorithm does not learn to diagnose; it learns to predict the chain of human events leading to a diagnosis [22]. AIs are thus more rightfully seen as “thinking partners” than replacements. Many participants in this study seemed to intuitively understand this: that a deep understanding of AI is required in order to be able to safely incorporate it into the operating theatre. Per participant 17, “If you’re not familiar with the process, how the data is created, then you can’t know what kind of errors can arise”. Likewise, participant 19 was concerned that “if you decide to go against that recommendation, then there’s kind of an ethical and moral dilemma, right? What if the patient then dies? Then you must ask, ‘What could I have done better?’ Should one always do the therapy recommended by artificial intelligence? So that’s kind of very difficult”. These statements reflect many of the discussion points found in the literature regarding the ethics and practical implementation of AI, for example, concerns regarding “explainability” and responsibility [35].

Nevertheless, participants in this study were generally, although not exclusively, positive, in both parts, about the use of AI in a predictive capacity. This is notable, given that AI is increasingly being applied in this fashion, whether to predict a difficult airway [36], intraoperative vital sign changes [8], or postoperative analgesia requirements [37]. However, an interesting subset of respondents (10 of 67, 15%) was skeptical about the role AI will play in the future in anesthesia. Participant 1, for example, when asked about using AI as a predictive technology, replied, “it is so extremely different, from patient to patient, that I can’t imagine that an AI can manage that”. Again, this mirrors previous work with clinicians on AI in medicine, who found that general practitioners largely considered the potential of AI in their field to be limited [38], as well as related literature on technology adoption, which frequently identifies a core of “active resisters” to new technology [16].

### 4.2. Comparison to Prior Work

In the existing literature, which consists almost exclusively of survey-based data, most studies have found that a plurality of physicians in a variety of specialties see the integration of AI into medicine as a positive development and that, the more technologically adept physicians are, the more positive their attitudes [9,10,11,12,39,40]. One of the only studies reporting a negative association, in which general practitioners in the UK were largely skeptical of the ability of AI to contribute meaningfully to their work, is also one of the only studies to utilize qualitative data [38]. This study extends these findings to the field of anesthesia, finding an overall strikingly positive response to closed questions but a rich collection of nuanced observations, both positive and negative, in interviews. On the basis of these data, it could be argued that these prior surveys have not captured the full range of clinicians’ thoughts and opinions regarding AI. Here, it is insightful to look at efforts to develop more comprehensive surveys, such as the new General Attitudes towards Artificial Intelligence Scale (GAAIS) from Schepman et al., which appears to better explore the full range of participants’ opinions [41].

### 4.3. Limitations

The data for this study were gathered using an inductive approach to the thematic analysis of interview data. These interviews were open-ended, standardized, and conducted by three different interviewers. This hypothesis-free-but-hypothesis-generating tactic, applied to a large dataset, lends credibility to these findings [42]. Furthermore, combining interview and survey data, on the one hand, yielded insights that either method alone would not have and, on the other hand, led to a perhaps somewhat leading survey, based as it was specifically on themes arising during the interviews.

However, it should be noted that participating anesthesiologists in both parts of the study trended younger and more female than the workforce at large. This was not intentional—and, indeed, the gender mix in the institutions where this study was carried out is roughly equal—but rather resulted from the relative availability of younger anesthesiologists in the course of the working day. In addition, age is a well-established moderator of attitudes toward new technology, with younger people generally more positively predisposed to new technologies. Finally, participants were recruited exclusively from university-affiliated hospital settings in which clinicians are arguably more used to trialing new technologies. All of this could perhaps explain the overall enthusiasm for AI in our cohort. Moreover, comparisons to similar cohorts are not possible, as this is the first such study in the field of anesthesia. Given this, further studies, especially as AI technology begins to be more widely adopted clinically, are warranted.

In addition, it should also be noted that the online survey, brief as it was, was not able to cover all the nuances in the data from the qualitative part of the study. Thus, the generalizability of many interesting points raised by our study participants remains unclear.

### 4.4. Conclusions

In this study of what anesthesiologists already know and think about AI, we have established that anesthesiologists appear to be generally well informed about AI and take a balanced view of the integration of AI in anesthesia. They are aware of some of the pitfalls of AI while being cautiously optimistic about, especially, the technical benefits it could bring to patients, above all in terms of its predictive capacity. Our results further suggest that there remains a small group of skeptics who will require a high degree of evidence to be “won over” to AI-based technologies. If AI is to successfully make the jump from research into the clinic, developers will need to build on clinicians’ pre-existing knowledge base and help practicing anesthesiologists navigate the strengths and weaknesses of this new technology to achieve successful implementation.

## Figures and Tables

**Table 1:** Participant characteristics in part one and part two of the study. Values are median (range (IQR)) or number (percentage). Participating centers were University Hospital Frankfurt (UKF), University Hospital Würzburg (UKW), and University Hospital Zurich (USZ).

Participant Characteristics	Part One: Interviews (*n* = 21)	Part Two: Survey (*n* = 49)
Age (y)	33 (26–55 [28–35])	34 (25–55 [28–37])
Male	5 (24%)	-
Female	16 (76%)	-
Experience (y)	3 (0–25 [2–8])	4 (1–26 [2–7])
Resident	17 (81%)	34 (69%)
Attending	4 (19%)	15 (31%)
Participating Center	UKF, UKW	UKF, UKW, USZ

**Table 2:** An overview of responses to questions 1 and 2, demonstrating a good pre-existing understanding of AI, with example statements. Values are number (percentage).

Question	Code	Statements	Example Statement(s)
What do you understand by the terms “artificial intelligence” or “machine learning”?	Little/no prior knowledge	4/90 (4%)	“I don’t really have a clue about that” (participant 16)
	Information technology	44/90 (49%)	“With the help of algorithms that people program … they train a system” (participant 7)
	Capabilities	34/90 (38%)	“Computers [which] adjust and perfect their predictions based on performance” (participant 3) “Computers learn new things themselves” (participant 4)
	Clinical support tool	8/90 (9%)	“Integrate computing into everyday clinical life” (participant 2)
Are you aware of any applications of artificial intelligence or machine learning in anesthesia?	Research	7/42 (17%)	“There’s a lot of research going on right now, and we’re also carrying out the ENVISION study, which uses AI” (participant 18)
	Other specialty	5/42 (12%)	“I’ve heard of it in radiology” (participant 13)
	Non-AI/-ML example	7/42 (17%)	“We have an EEG monitor. I assume that this is relevant” (participant 13)
	None	23/42 (55%)	“I can’t think of anything” (participant 10)

**Table 3:** An overview of responses to question 3, demonstrating a balanced view of the pros and cons of AI as applied to anesthesia, with example statements. Values are number (percentage).

Question	Code	Number	Example Statement(s)
What do you think are the advantages and disadvantages of artificial intelligence in anesthesia?	Technical pros	24/105 (23%)	“Machines don’t get tired, they don’t have bad days, they usually function better, they have a better memory than any human being and they have an unlimited capacity for learning” (participant 8) “We rely a lot on experience, on feeling—and that is sometimes justified—but I think that in some areas, artificial intelligence is more precise and perhaps makes better decisions than humans” (participant 20)
	Human-computer interaction pros	22/105 (21%)	“It can support you in making decisions, especially when you are uncertain, [for example when] parameters are perhaps contradictory” [participant 20]
	Technical cons	21/105 (20%)	“The situation can be diverse or much more differentiated than an algorithm can reckon with” (participant 2) “In medicine … there is often a gender bias, especially in drug studies, and that is a challenge to overcome” (participant 18)
	Human-computer interaction cons	38/105 (36%)	“The anesthesiologist can now spontaneously decide ‘okay, I’ll give fentanyl now’ and the AI only knows that when it is given as an input” [participant 6] “If the system fails, you are left standing there and no longer know how things work” (participant 12) “If you decide to go against that recommendation, then there’s kind of an ethical and moral dilemma, right? What if the patient then dies? Then you must ask ‘What could I have done better?’ Should one always do the therapy recommended by artificial intelligence? So that’s kind of very difficult”. (participant 19)

**Table 4:** An overview of responses to questions 4 and 5, demonstrating a positive view of the use of AI to predict important clinical events, with example statements. Values are number (percentage).

Question	Code	Number	Example Statement(s)
In particular, what do you think about the use of artificial intelligence to make predictions?	Positive statement	32/67 (48%)	“You could prepare yourself better for situations, with fewer surprises, which probably also means better patient safety” (participant 10)
	Negative statement	6/67 (9%)	“You perhaps lose the bigger picture a little bit” (participant 5)
	Cautious/neutral statement	19/67 (28%)	“So long as there’s still someone behind it who interprets it or what it means and reacts properly to it” (participant 13)
	Not possible/technology too immature	10/67 (15%)	“Things are so extremely different from patient to patient that I can’t imagine that an AI can manage that” (participant 1)
Which predictions do you think are most useful clinically?	Risk stratification	14/92 (15%)	“Outcome relevant points. [For example] Is the patient at risk of PONV, postoperative myocardial infarction, stroke, etc.?” (participant 7)
	Vital sign predictions	36/92 (39%)	“The viral parameters, of course” (participant 6)
	Treatment guide	17/92 (18%)	“I think what I have not yet seen was the post-operative analgesic requirement, which would certainly be exciting” (participant 13)
	Not useful/technologically impossible	6/92 (7%)	“If that’s even possible, to predict like that” (participant 5)
	Event type	19/92 (21%)	“Acute events in the operating theatre” (participant 7) “Things that are quite subtle, and that could easily be overlooked” (participant 20)

**Figure A1:**
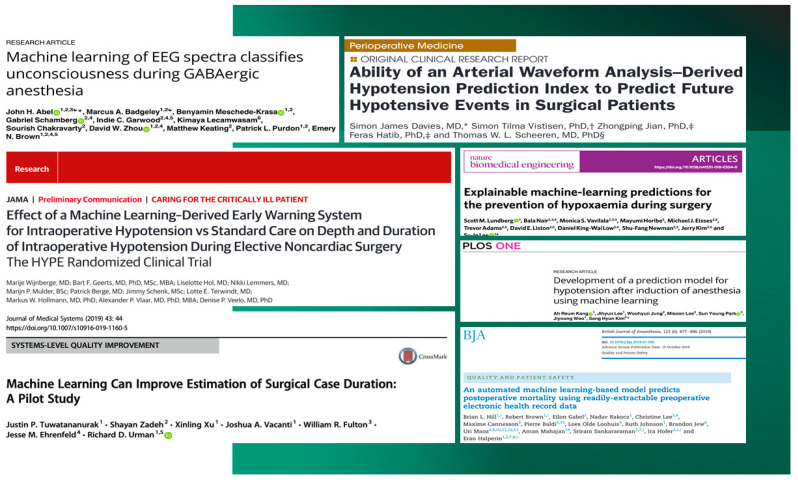
Collection of recent examples of applications of artificial intelligence to anesthesia, shown to interviewees after question 2.

## Data Availability

All data are available in the appendices to this paper.

## Appendix A. Interview Questions

English Translation

1. What do you understand by the terms “artificial intelligence” or “machine learning”?

After question 1, study participants were provided with the following definitions of artificial intelligence and machine learning: (English) “Artificial intelligence is a branch of computer science that deals with the automation of intelligent behaviour. Machine learning is a sub-area of artificial intelligence.” (German) «Künstliche Intelligenz ist ein Teilgebiet der Informatik, das sich mit der Automatisierung von intelligentem Verhalten befasst. Maschinelles Lernen ist ein Teilbereich der künstlichen Intelligenz.»

2. Are you aware of any applications of artificial intelligence or machine learning in anesthesia?

After question 2, study participants were shown a collection of recent examples of applications of artificial intelligence to anesthesia (see Figure A1).

3. What do you think are the advantages and disadvantages of artificial intelligence in anesthesia?
4. In particular, what do you think about the use of artificial intelligence to make predictions?
5. Which predictions do you think are most useful clinically?

German Translation

1. Was verstehen Sie unter den Begriffen «künstliche Intelligenz» oder «maschinelles Lernen»?
2. Sind Ihnen Anwendungen von künstlicher Intelligenz in der Anästhesie bekannt?
3. Welche Vor- und Nachteile könnte die künstliche Intelligenz in der Anästhesie Ihrer Meinung nach haben?
4. Insbesondere, was halten Sie von der Verwendung künstlicher Intelligenz zur Erstellung von Vorhersagen?
5. Welche Vorhersagen («Predictions») sind Ihrer Meinung nach besonders nützlich?

## Appendix B. Coding Schemes

**Table A1:** Coding schemes derived from interview statements in part 1 of the study.

1.1 Little/no prior knowledge
1.2 Information technology
1.3 Capabilities
1.4 Clinical support tool
2.1 Clinical
2.2 Research
2.3 Other specialty
2.4 Non-AI/-ML example
2.5 None
3.1 Pros
3.1.1 Technical pros
3.1.2 Human–computer interaction pros
3.2 Cons
3.2.1 Technical cons
3.2.2 Human–computer interaction cons
4.1 Positive statement
4.2 Negative statement
4.3 Cautious/neutral statement
4.4 Not possible/technology too immature
5.1 Risk stratification
5.2 Vital sign prediction
5.3 Treatment guide
5.4 Not useful/technologically impossible
5.5 Event type

## Appendix C. Online Survey

English Translation

Statement 1: I have a working/general understanding of what artificial intelligence is

Statement 2: I don’t currently use any technology based on artificial intelligence/machine learning at work

Statement 3: I think applying artificial intelligence to anesthesia will bring mostly benefits

Statement 4: It is important to me to understand the technologies I use in the operating room

Statement 5: Predicting clinically meaningful events (e.g., hypotension) before they occur would be helpful to me in my work

German Translation

Statement 1: Ich habe eine allgemeine Vorstellung davon, was künstliche Intelligenz ist

Statement 2: Ich verwende derzeit bei der Arbeit keine Technologie, die auf künstlicher Intelligenz/Maschinenlernen basiert

Statement 3: Ich denke, dass die Anwendung künstlicher Intelligenz in der Anästhesie vor allem von Vorteil sein wird

Statement 4: Es ist wichtig für mich, die Geräte zu verstehen, die ich im Operationssaal verwende

Statement 5: Die Vorhersage klinisch bedeutsamer Ereignisse (z. B. Hypotonie), bevor sie eintreten, würde mir helfen, die Patienten besser zu betreuen

## Appendix D. Interview Transcripts

Participant 1

1. What do you understand by the terms “artificial intelligence” or “machine learning”?

English: “Artificial intelligence is when a... the [programme] can [combine the information it receives] and [draw conclusions].”

2. Are you aware of applications of artificial intelligence in anaesthesia?

English: “[No, not really].”

3. What do you think are the advantages and disadvantages of artificial intelligence in anaesthesia?

English: “The advantage is certainly a [supportive instrument to prevent possible dangers]. Disadvantages? [I would not rely on that]. In case of doubt, [I only rely on myself]. Because [just because the AI doesn’t foresee something doesn’t mean it can’t happen].”

4. In particular, what do you think about the use of artificial intelligence to make predictions?

English: “It’s actually the case that [everything is so individually different that you need to train a separate AI for each operating theatre team]. There’s a nurse who instruments differently or has a language barrier. There is the surgeon who is having a bad day today. So [there are many, many parameters that are actually different from one operation to the next, but still have a massive effect on the outcome]. [It is so extremely different from patient to patient that I can’t imagine that an AI can manage that]. I had two identical patients back last week. One had hypotension after induction and the other did not. So... [I don’t think these things are predictable].”

5. Which predictions do you think are most useful clinically?

English: “The [EEG prediction regarding wakefulness] and the prediction regarding [relaxation time]. This could ultimately help to plan the anaesthesia a little better. The prediction of heart rate or blood pressure... [I wouldn’t want heart rate now], but [low blood pressure would certainly be good if one could predict that].”

Participant 2

1. What do you understand by the terms “artificial intelligence” or “machine learning”?

English: “[Computer systems] that [help you to understand things more easily] or to [integrate computing into everyday clinical life] or everyday life in general.”

2. Are you aware of applications of artificial intelligence in anaesthesia?

English: “[Not much so far]. [No, none]. [At least none that we would use].”

3. What do you think are the advantages and disadvantages of artificial intelligence in anaesthesia?

English: “In order [to have an easier overview of facts or things], i.e., [to be able to filter more quickly]? Also [in terms of patient safety, that certain things that you might not recognise right away can be recognised more easily through AI]. That [in the end it is still just a computer that works according to algorithms]. [The patient often various aspects not recognised by the AI system, which the algorithms overlook], or [which simply cannot be programmed in]. Yes, that is certainly the case [with] algorithms. [Humans also make mistakes that an algorithm might recognise more easily], but at the same time, [that’s probably the biggest weakness, that there’s also the exception that algorithms don’t recognise], because [the situation can be diverse or much more differentiated than an algorithm can reckon with]. And [there are several variables that are not recognisable with the algorithm].”

4. In particular, what do you think about the use of artificial intelligence to make predictions?

English: “But [that is always difficult, the physiological consequences of administering medication].”

5. Which predictions do you think are most useful clinically?

English: “For example, [systems that allow you to categorise patients]. For example, [the perioperative risk], [so that you can filter out patients who have a high cardiac risk, for example, without having to think too much beforehand]. For example, [you can read off cardiac risks, for example with no idea of heart failure, how is it graduated, that you make a scoring system]. [Monitoring is also a way of visualising things that could happen], of course, but also [after administering a medication, perhaps taking its effects into account].”

Participant 3

1. What do you understand by the terms “artificial intelligence” or “machine learning”?

English: “That [computers learn new things themselves] [based on existing data], particularly for machine learning, that they [predict things themselves] and then also [adjust and perfect their predictions based on experience]. And that is based on artificial intelligence.”

2. Are you aware of applications of artificial intelligence in anaesthesia?

English: “Not in the clinic. [In research it is for various predictions]. There are also [models that you either select clinically through algorithms, that is clinically through regression, or through some kind of tree... map, I think it’s called, where you let it select through that], but I know it so far, it hasn’t convinced me yet, because it has always performed worse than if you have selected things clinically beforehand and you have tested them.”

3. What do you think are the advantages and disadvantages of artificial intelligence in anaesthesia?

English: “I can imagine that in some cases, [because you have so many parameters, it’s an advantage], because [there are many parameters where a person might need a few learning cases before he can predict as well as the AI], that it’s better. But I can imagine a disadvantage where in other things, [that some things are preferred in error, where you as a doctor just know, no, that’s...] For example, when we measure wakefulness now, we have a device that works better now, but is still often wrong. And then I throw that out of my thinking completely, for example, because [I know that other parameters are more important and that it could be that it turns out differently].”

4. In particular, what do you think about the use of artificial intelligence to make predictions?

English: “I think [it’s good if it improves in the future]. As I said, [we tried this once with a stroke prediction score. It didn’t work at all]. Although we had many patients, a large amount of data. But [I think it’s good in combination, as a tool]. [I think it can also be good], but [in the end it should always be evaluated by a doctor or a person who should control it]. [Maybe in the future it will get better and better].”

5. Which predictions do you think are most useful clinically?

English: “[Complications, post-op, also intra-op], if there are really any parameters, like maybe [hypotension] or something like that, where there are [more [or] less obvious things that might become important in combination]. Something like [PPV] or other things, something like [the patient will not be relaxed] anymore, I mean, you can also read the TOF, then you can also see the trend. So that depends. But for that, for [complications] or something, then maybe. Or if there is a [certain trend that the prediction models see faster than we do]. Like, for example, the [blood pressure is going to crash]. The pressure [will be low on the basis of things that the models notice earlier than we see the trend, so by the time we notice it, it’s maybe already too late], so it’s already low, maybe something like that.”

Participant 4

1. What do you understand by the terms “artificial intelligence” or “machine learning”?

English: “In principle, [a system] that [continues to learn from itself] and [replaces humans] in the sense that it [continues to learn from itself] [on the basis of an algorithm], [can think ahead independently] and [can predict things].”

2. Are you aware of applications of artificial intelligence in anaesthesia?

English: “In anaesthesia? Um, [no, not really], I would say now.”

3. What do you think are the advantages and disadvantages of artificial intelligence in anaesthesia?

English: “I think a big advantage is that [it can bridge different levels of anaesthesiologist knowledge]. So [someone who has little experience is certainly more supported than someone who already has this clinical experience]. On the other hand, I could imagine that [it also prevents you from thinking for yourself, because something else is already doing that]. I think that would be two big advantages and disadvantages that I can imagine.”

4. In particular, what do you think about the use of artificial intelligence to make predictions?

English: “[I do believe that it can be helpful], [especially when it comes to acute deterioration], [to be forewarned], so that [you can calmly think about what to do and how I can change it. Can I get some kind of help in good time]? But that’s where it makes sense. Yes, that’s right.”

5. Which predictions do you think are most useful clinically?

English: “Especially somehow [airway management], I think [temperature things that are not so acute, it makes less of a difference]. Probably. But I think it’s [acute deteriorations] that you could predict.”

Participant 5

1. What do you understand by the terms “artificial intelligence” or “machine learning”?

English: “Maybe, [machine-generated reasoning instead of human reasoning]?”

2. Are you aware of applications of artificial intelligence in anaesthesia?

English: “[Not with us], [not anymore].”

3. What do you think are the advantages and disadvantages of artificial intelligence in anaesthesia?

English: “The advantage is probably that [it is more widely applicable]. The disadvantage is probably... [it could be that one is pushed in a given direction] [and no longer thinks about it so freely]. It helps, of course, because [you can react to events more quickly if they can be predicted] well. I think that’s great. So if that works or would work in everyday life, it would of course be brilliant. [I just wonder if it really works in such a way that you can foresee it], because then you already have a chance to react to it before it happens. So that would be ingenious, of course.”

4. In particular, what do you think about the use of artificial intelligence to make predictions?

English: “It’s probably the same issue, that [you’re actually just always waiting to see what changes and perhaps lose the bigger picture a little bit] when you have the patient in front of you like this. But of course [that can also be an advantage]. But [I would see that as a danger], that [one is a bit too focused on something specific changing now and no longer looks at the overall picture].”

5. Which predictions do you think are most useful clinically?

English: “Yes, basically almost all of them, but I mean, for example, [hypothermia is readily avoidable], or [hypovolaemia]... If I could predict it, I can of course avert it more quickly, [if that’s even possible, to predict like that]. That is... yes, the becoming would be the most important thing, i.e., [the avoidable events]. I mean, if I say now that he will have an [tachycardia later on, I can’t treat that beforehand, that has some reason, I probably can’t treat it so well beforehand], in inverted commas. But if I have [hypovolaemia] or [hypothermia], then I can react beforehand.”

Participant 6

1. What do you understand by the terms “artificial intelligence” or “machine learning”?

English: “Machine learning today? You have [a neural network with different nodes, which have input and output and the output can then be passed on to several nodes at the next level. And depending on how this output is weighted, i.e., how the input is amplified or degraded and passed on], a neural network can then [make decisions] afterwards, depending on how it was set up.”

2. Are you aware of applications of artificial intelligence in anaesthesia?

English: “There’s bound to be something done in anaesthesia [I don’t know yet].”

3. What do you think are the advantages and disadvantages of artificial intelligence in anaesthesia?

English: “Well, in general, artificial intelligence [is only as good as the material with which it has been trained]. And here [you probably need a lot of input to eventually get a reasonable output]. And then it’s also the case that [if you hypothetically let an artificial intelligence make predictions about anaesthesia, we humans also play a role as disruptive factors]. Because [the anaesthetist can now spontaneously decide okay, I’ll give fentanyl now and the AI only knows that when it is given as an input]. I can imagine that [when you give a drug at that moment, reality takes a completely different direction than the prediction of artificial intelligence].”

4. In particular, what do you think about the use of artificial intelligence to make predictions?

English: “As we have already said, [we need an input and the output is only as good as the input]. [I would like that, I think it makes sense]. But [you have to be very aware of the device and how it works], and [there will certainly be many more attempts before this is established and used sensibly].”

5. Which predictions do you think are most useful clinically?

English: “[Depth of anaesthesia] measurements are useful, I have also done experiments with propofol and prediction, because as far as I know it was not AI, but simply a computer model that then made predictions based on patent-specific variables. [If you could do that better with artificial intelligence [propofol dosing], then it would be a big step forward]. In the same way, [artificial intelligence could also predict: what are the opiate levels, whether I should perhaps give analgesia again]. And clearly [include the vital parameters of course].”

Participant 7

1. What do you understand by the terms “artificial intelligence” or “machine learning”?

English: “It’s quite interesting, I’m also studying it myself. And what I understand by that is that [with the help of algorithms that people program], which I don’t understand that much about, they train a system]. And [they teach the system] to [make decisions for us] or for the doctors and, in particular, [to optimise and improve care].”

2. Are you aware of applications of artificial intelligence in anaesthesia?

English: “So I don’t know at all whether this is actually artificial intelligence, that is, [decision-making, support systems and decision-making aids are widespread in medicine in anaesthesia]... what is really done with artificial intelligence... [I’m not personally aware of anything], but it could also be my personal ignorance. Well, I only know that [at least in radiology it has been done more often], that the artificial intelligence of algorithms... we want that, but... we are in the process, but I...”

3. What do you think are the advantages and disadvantages of artificial intelligence in anaesthesia?

English: “Well, the advantage is that [a machine learns things more often], if you can put it that way, and through these experiences and [can therefore make more informed decisions] and perhaps also with less stress. But of course [it’s also a computer-based learning, which may be error-prone or may have errors in the learning process] or [may be subject to some biases]. [Of course, one can always say that the human being may still be able to assess more all-encompassing capabilities with experience in a different way.]. And I think [we are also often totally biased as a result of our experiences]. For example, in the case that you have a patient who is for some reason tachycardic and hypotensive: one person sees a haemorrhage, and the next sepsis, and the next, a surgeon, doesn’t want to admit there’s anything wrong at all. So [everyone is somehow subject to their’ own personal relationship and their previous experiences]. And not the now... And [that bias comes into play with us, but can be filtered out by technology or by the computer].”

4. In particular, what do you think about the use of artificial intelligence to make predictions?

English: “Yes, a lot. So that [in itself is something totally innovative] and I think [still too little used in medicine], because we are all relative beginners in the profession and we also learn a great deal from experience and from situations. That was the case in such and such a situation, especially in anaesthesia... that’s the only thing that happens. You have a direct consequence from an action and [from algorithms that show you something, they are much cleverer than humans and faster and probably more reliable if they have already gone through it millions of times in their computer]. [We need years for that, maybe to achieve what the machine achieves]. But [I think it’s very difficult to train it well with data]. I could imagine. So with [patient data or surgery data, it’s already difficult to integrate that into a machine learning programme and have it learn well]. So I can imagine... because [the interface between technology and informatics and medicine is already a big hurdle]. So similar in our project and data protection, the law. [In itself, it’s a super, super good idea] and [I think it can take us a long way and maybe also in medicine], but [it still needs a a while until it’s ready].”

5. Which predictions do you think are most useful clinically?

English: “Well, starting with [acute predictions]. So [if I give this drug and that drug, what happens to heart rate, blood pressure etc.] but also [outcome relevant points. Is the patient at risk of PONV and postoperative myocardial infarction, stroke, etc.?] There are many, many risk scores and predictions, but they are not in our everyday life and maybe you do it better than we do, but in anaesthesia you still sit in your pre-med [room] and look at a patient and think hmm he is sick, he is healthy. But [if you could somehow calculate that there is such and such a high risk of suffering a heart attack or such a high risk and that you have to pay special attention to this on the one hand and also have ways of avoiding this if you know that there is perhaps a higher probability that this will occur], that I think that it is [the acute events in the operating theatre], whereby [it is of course already clear that if I inject someone with 200 propofol that he will then become hypotensive]. Maybe I don’t need a prediction, but [maybe there are major complications, and I think that’s very relevant]. Yes.”

Participant 8

1. What do you understand by the terms “artificial intelligence” or “machine learning”?

English: “For me, artificial intelligence is [a computer system] that [can learn on its own]. It has some specifications [programmed by humans], but [is then able to learn on its own], without humans having to [feed it information] or [provide any algorithms] with which [it then learns]. Or maybe someone can even develop [self-developing algorithms] and develop them further that way.”

2. Are you aware of applications of artificial intelligence in anaesthesia?

English: “[Not yet, to be honest], but [it will exist somewhere, I think, running in the background] But [I don’t know now].”

3. What do you think are the advantages and disadvantages of artificial intelligence in anaesthesia?

English: “I think one disadvantage is [coming to rely too much on machines]. [There are always potential sources of error, whether from the user or something being transmitted incorrectly, the machine can also be wrong]. And I believe that [if you rely too much on machines, at some point, there is a danger that you no longer look at the patient yourself]. For example, the device may say the patient is asleep, when that may not be the case. But I see more advantages than disadvantages, because [AI and ML] work with algorithms. [Machines don’t get tired, they don’t have bad days, they usually function better, they have a better memory than any human being and they have an unlimited capacity for learning] and can support us in many decisions. [Maybe not in taking decisions, but in many situations, computers can support us], because algorithms are simply logical paths that you work through one after the other, which is what you do in everyday life, but of course [as a human being you are much more prone to error than a machine]. [So, ultimately, I think these technologies are more useful than harmful].”

4. In particular, what do you think about the use of artificial intelligence to make predictions?

English: “[I think it would probably be good for standard situations]. But, given our line of work, [I’m somewhat sceptical, because sometimes unexpected things happen. For example, during an operation the surgeon unexpectedly cuts into a large blood vessel. The computer certainly can’t predict that because it just happens]. Um, [with other things, events that develop over a longer period on the intensive care unit, for example, I think that it could probably be useful]. In the operating theatre, I don’t know exactly, [if you’re busy with other tasks at the same time, then maybe it can provide a warning]. But [you can set the alarm limits a bit tighter for a lot of things and thus achieve the same thing]. So especially in acute situations, where the patient’s condition changes relatively quickly simply depending on the environment, the operation, I find difficult. I can well imagine that, but [for other things, where you have the patient for a longer period and there is relatively little influence on him/her from the outside, I do believe that it can help]. [I think it depends on the situation the patient is in].”

5. Which predictions do you think are most useful clinically?

English: “During anaesthesia, [depth of anaesthesia], so that I don’t give too little anaesthetic medication. That’s a bit of a black box where who knows what happens. One might not always recognise that in good time. Otherwise, [blood pressure] is extremely dependent on the substances I administer, especially during anaesthesia. And if I were perhaps more inexperienced, because it’s really difficult to tell whether the patient will become [hypoxic], perhaps it would be good if the computer could look at [respiratory rate]. Before I look at the patient after extubation, for example, [the computer could recognise in time, okay, he’s not breathing, while I’m busy with some documentation or something, it could give me a warning signal] before the [saturations] starts to drop. That might be something useful. And as I said, [in the intensive care unit I also predictions could be helpful. Does the patient still need volume? Or doesn’t he?] That something like that is picked up automatically, I think that would also be quite useful. [Or does he need an EC or not?] If that could be guided by an algorithm, it might help me in the decision-making process, something like that.”

Participant 9

1. What do you understand by the terms “artificial intelligence” or “machine learning”?

English: “Yes, ultimately it is more. I would say that [data is evaluated] [via an algorithm], which makes it easier [to make certain predictions] or [optimise things]. But it’s precisely [from large amounts of data].”

2. Are you aware of applications of artificial intelligence in anaesthesia?

English: “So I know that [our anaesthesia machine can definitely make a prediction of the Mac, whether that’s via artificial intelligence. probably it’s a simply a mathematical formula]. I think that’s overstating it. [In anaesthesia I don’t know]. No... [in research. I know that, for example, they do image analysis here in pain research using artificial intelligence]. But [I don’t know of anything being used clinically].”

3. What do you think are the advantages and disadvantages of artificial intelligence in anaesthesia?

English: “So the advantages are definitely that [there is finally a system that can evaluate the vast amounts of data that we are currently recording digitally], via anaesthesia protocols, where it is documented which drug is given when, which patient it is, how the vital parameters are, and finally with a large data set and then [make predictions]. That is of course super good. I would almost say that anaesthesia is almost only advantages in other areas. After all, [we have interpersonal contact. It can’t be calculated away, of course, and of course an artificial intelligence can’t do that]. But apart from that, I think there has to be much, much more. Yes, in my head there are only advantages. I always say that for learning, too. [Certain empirical knowledge comes from experience, of course, that will always be the case. But AI can also help you to understand a certain experience, to see certain things developing. So that, even when you don’t have the system, you might think the AI has always warned me in this case, or made me aware of certain side effects. And so on. That that can have a certain teaching effect]. That’s why I don’t think there are any disadvantages in this area.”

4. In particular, what do you think about the use of artificial intelligence to make predictions?

English: “I think [it can definitely give a hint]. [Whether the hint is actually true or the prediction gives a clear indication to act is questionable]. So if the doctor says, okay, after giving a certain medication the heart rate or the blood pressure will drop with medium-urgency and I should then give this medication as a preventive measure. [I don’t know if that’s a clear indication, but you can see in everyday life that there are people who do something preventive relatively early, before you see anything]. And I think that’s what it’s all about. [You shouldn’t take it for a value that is, let’s say, final and that’s how it’s done. Rather, it should ultimately provide a bit of support, an impetus to say that I have to consider that this could happen]. And that’s exactly how I would see it. [Just as a support, as a back up].”

5. Which predictions do you think are most useful clinically?

English: “The classic question about [relaxation], of course. That would be nice, of course, but in the end it doesn’t make any difference any more. If the surgeon says it’s too tight, then it’s too tight. No matter what my prediction is. [The blood pressure] is a great thing, of course, even if you. Yes, that. Yes, that would be nice, of course. But [I don’t know whether it works so well that you actually have a valid statement in complex cases or whether you have a gut feeling and say, well, I’ll have to see whether that’s really the most sensible thing to do]. [I think it’s good if it’s an overall package where you’re nudged a little bit everywhere. But I don’t have in mind that I say, here in this case it’s essential].”

Participant 10

1. What do you understand by the terms “artificial intelligence” or “machine learning”?

English: “On the one hand, I associate it with the fact that [it is very complex, rather difficult to somehow understand and navigate]. But on the other hand, I also think about [saving time and perhaps also minimising errors].”

2. Are you aware of applications of artificial intelligence in anaesthesia?

English: “[Nothing directly]. [I can’t think of anything].”

3. What do you think are the advantages and disadvantages of artificial intelligence in anaesthesia?

English: “A disadvantage, perhaps, is that [you might rely too much on it] and [think less for yourself or think ahead less]. Um yes. Advantages. It’s [less user dependent. In other words, you might recognise something faster than somebody else] … [it might also make fewer mistakes]? This would [improve safety and save time for some things].”

4. In particular, what do you think about the use of artificial intelligence to make predictions?

English: “Yes, [I think it’s a good idea]. Maybe then [you could prepare yourself better for situations, with fewer surprises, which probably also means better patient safety. So, I think it’s actually very good].”

5. Which predictions do you think are most useful clinically?

English: “Maybe something like [hypotension], maybe something like [difficult airway management] or something like that, maybe also something like [blood loss, transfusion] or something like that. Well, [maybe just everywhere where it often gets stressful when things unexpectedly turn out differently than you had planned].”

Participant 11

1. What do you understand by the terms “artificial intelligence” or “machine learning”?

English: “By artificial intelligence and machine learning, I understand that [a computer scans pathologies according to predefined parameters and highlights a range of diagnoses and differential diagnoses accordingly].”

2. Are you aware of applications of artificial intelligence in anaesthesia?

English: “[No, honestly not]. Not consciously, anyway. No, not with us. But I haven’t been in the operating theatre for a long time. It could just be that it’s because of that.”

3. What do you think are the advantages and disadvantages of artificial intelligence in anaesthesia?

English: “[Advantages are safety, that sometimes you don’t notice something because you’re busy with other things on the patient, repositioning or something else, that something is just shown]. But one disadvantage is perhaps that [you then switch off your head and simply rely on the PC or the programme] and [perhaps at some point you can no longer really understand the pathophysiology]. Or [the forward thinking that you need as an anaesthetist can be lost].”

4. In particular, what do you think about the use of artificial intelligence to make predictions?

English: “[I think that’s good, because you could then prepare oneself accordingly for what needs to be done, and not be caught unawares].”

5. Which predictions do you think are most useful clinically?

English: “Anaesthesia is such an [acute emergency subject and as I said, if you just somehow knew that a step beforehand, then you can intervene much earlier], really prepare for it and that is certainly better for patient care, which simply makes patients safer.”

Participant 12

1. What do you understand by the terms “artificial intelligence” or “machine learning”?

English: “Well, it’s something [relatively futuristic], [which I don’t spontaneously associate with any particular concept], to be honest. I understand [something with computers], in any case [something where you need a lot of understanding of technology]. But also [something about the future]. You [can probably make many areas simpler or structure them more clearly [using AI/ML]].”

2. Are you aware of applications of artificial intelligence in anaesthesia?

English: “At least [in our clinic, I don’t know if I would call anything artificial intelligence]. Otherwise, I know that there have been efforts in our clinic recently [to link certain decision-making processes and/or certain ways of economising a bit more with artificial intelligence]. The idea is that you can fall back on it in your decisions. But I can’t be more specific about that.”

3. What do you think are the advantages and disadvantages of artificial intelligence in anaesthesia?

English: “An advantage is, of course, that [events could be predicted, and this could improve patient safety]. Simply put, [you are less dependent on human error], that [standard events are foreseen and can [therefore] be prevented]. But on the other hand, [artificial intelligence or machine learning may not be as good at predicting unforeseen events as humans]. Of course, this could just be optimism on my part, but [you could come to rely too much on the computer telling you that a patient will become hypotensive and perhaps no longer be so aware yourself]. So, [you could come to think too little], [rely too much on that]. Or [if the system fails, that you are left standing there and no longer know how things work]. I know, for example, that there was an artificial intelligence project or a company that wanted to establish such a device for nociception and that’s also something that you learn to evaluate yourself, whether a patient is in pain during the operation or at least under stress, and when that is more or less taken away from you, [you then rely on values and no longer interpret other vital parameters yourself], with which you perhaps could arrive at the same result much more quickly.”

4. In particular, what do you think about the use of artificial intelligence to make predictions?

English: “[I think it is an excellent additional tool, especially for beginners and young colleagues to gain a bit more confidence through it]. [You just have to be careful not to rely purely on it], [I think it could be dangerous when used alone, because it can’t read in any other way].”

5. Which predictions do you think are most useful clinically?

English: “[Everything that can potentially endanger the patient], in other words, everything that can cause harm to the patient or make harm more likely, is of course a good thing to have.”

Participant 13

1. What do you understand by the terms “artificial intelligence” or “machine learning”?

English: “Machine learning is learning [on the basis of big data] and, in contrast to medicine, where [you always have a causality somewhere, in machine learning, you simply have correlations] from the [volume of data], which you may not be able to store at all! And artificial intelligence is either simply machine learning with [very, very, very, very large amounts of data] or a little less technical but the application of data and [the evaluation of data] [with the help of algorithms] or machine learning.”

2. Are you aware of applications of artificial intelligence in anaesthesia?

English: “[I’ve heard of it in radiology]. In anaesthesia... [we now have Narcotrend, i.e., cerebral state index, a depth-of-anaesthesia measurement. I assume that this is relevant]. Otherwise I don’t think I can think of... [I think there is also anaesthesia prediction]. I would somehow summarise that under the topic, but that’s all I can think of now.”

3. What do you think are the advantages and disadvantages of artificial intelligence in anaesthesia?

English: “[It is likely to capture more nuance] simply through artificial intelligence and machine learning. One disadvantage is [perhaps that in some cases the causality is not known, and the anaesthetist may not know how to react to it because he only knows how a vital sign might change, but not why it is changing. For example, hypertension is predicted, but it is not clear why it will happen]. Maybe.”

4. In particular, what do you think about the use of artificial intelligence to make predictions?

English: “Um, [I think it’s good as an additional tool], [so long as it remains an aid and not a substitute, a way of flagging a problem, but then it’s still acted upon by someone with the academic background knowledge, so to speak, and it’s not simply flatly accepted]. [So long as there’s still someone behind it who interprets it or what it means and reacts properly to it].”

5. Which predictions do you think are most useful clinically?

English: “The need for [post-operative analgesia], I think, would be quite interesting. [Anaesthetic depth measurement] is a useful tool, but if that could be improved, that would also be good. And yes, of course, the prediction of intraoperative incidents such as [hypotension] or [hypoxaemia], would also certainly be good, to be warned in advance, so that you can remain vigilant and perhaps know that during this anaesthetic I have to pay a little more attention, but [I think what I have not yet seen was the post-operative analgesic requirement, which would certainly be exciting].”

Participant 14

1. What do you understand by the terms “artificial intelligence” or “machine learning”?

English: “So under artificial intelligence? I basically understand [a computer or some other kind of electronic device] that is [able to evaluate data] and [make a forecast from it]. In the end, that’s exactly what it is, i.e., [the creation of a forecast for the future] [based on a data set] that [the computer] has, which [the human brain could also do, but specifically from the computer]. Yes.”

2. Are you aware of applications of artificial intelligence in anaesthesia?

English: “In anaesthesia now less than [in radiology, for example]. So now in the clinic it’s not clear. Our [Perseus can make a respiratory gas prediction, that is perhaps also a lower, lower artificial intelligence], but otherwise [I can’t think of anything].”

3. What do you think are the advantages and disadvantages of artificial intelligence in anaesthesia?

English: “The advantage is that it is [less distorted by emotions or personal prejudices], especially with regard to the duration of the operation, for example, that it is [really only based on rational information]. The disadvantage is that [perhaps some human factors are not taken into account by the computer], that an operation at 3 a.m. always takes longer than during the day.”

4. In particular, what do you think about the use of artificial intelligence to make predictions?

English: “[I actually think it’s good]. Also [because you sometimes find yourself in the situation when an operation has been going on for three hours, the patient is completely stable, you have set the alarms and pay relatively little attention to anaesthesia, and it’s nice to have a prediction every now and then, like “hey, I have to do something again now”]. Because the patient is about to wake up or something.”

5. Which predictions do you think are most useful clinically?

English: “[Patient is waking up], which usually doesn’t work for me. We have a Narcotrend. If it tells me that the patient is awake, then the patient will be awake in ten minutes. Yes, also [things like ST segment], things like that, I don’t necessarily find at first glance in our one-line ECG. [Blood pressure] too. Those are the most important ones now.”

Participant 15

1. What do you understand by the terms “artificial intelligence” or “machine learning”?

English: “By artificial intelligence I understand [[computer-based] learning] by and of a computer [and algorithms], that then also basically [continue to learn how to apply these algorithms]?”

2. Are you aware of applications of artificial intelligence in anaesthesia?

English: “[Not really].”

3. What do you think are the advantages and disadvantages of artificial intelligence in anaesthesia?

English: “[An advantage is Being able to predict for example blood pressure, that a patient will become unstable]. A disadvantage is, of course, that [you might rely a little too much on being warned and no longer pay so much attention yourself].”

4. In particular, what do you think about the use of artificial intelligence to make predictions?
5. Which predictions do you think are most useful clinically?

English: “I think that it would be helpful to be warned a little in advance if, for example, there is too much [blood loss] and the patient is likely to become [unstable], that kind of thing. On the subject of [hypovolaemia], I can really imagine a use for predicting [volume management].”

Participant 16

1. What do you understand by the terms “artificial intelligence” or “machine learning”?

English: “My understanding of artificial intelligence is that [one collects data and uses this data] to [make predictions] to [improve patient care]. What was the other one? [“maschinelles Lernen”/”machine learning”] [I don’t really have a clue about that], to be honest. [I’ve never heard of it before].»

2. Are you aware of applications of artificial intelligence in anaesthesia?

English: “[I haven’t been on the intensive care unit here myself, but I know that we use it there], but I’ve never used it myself. So I can’t say too much more.”

3. What do you think are the advantages and disadvantages of artificial intelligence in anaesthesia?

English: “Well, with access to a large data set, then of course [your anaesthetic can be optimised]. [It can improve patient care]. I take a positive view of this because [we are only human, and we find things stressful sometimes. An AI, however, doesn’t miss things]. But of course, [there are always technical problems], just as [there can always be human error]. That’s also clear, of course. But basically, I see it very, very positively.”

4. In particular, what do you think about the use of artificial intelligence to make predictions?
5. Which predictions do you think are most useful clinically?

English: “So what I’ve had a lot to do with you lately is about [relaxation]. That was also presented in this patient’s case, [that you have continuous monitoring and that you can adapt it a bit to the course of the disease]. I think that’s great, because I see it very often in everyday life that things don’t run optimally for some reason, that you then paralyse in a way where you shouldn’t have paralysed at all, or too much, too little. And I find that extremely cool. I think that’s very relevant. But [EEG anaesthetic depth measurement] is also important. Especially for older patients.”

Participant 17

1. What do you understand by the terms “artificial intelligence” or “machine learning”?

English: “Probably [computer programmes] that [make themselves smarter], that is, [they learn] [through certain algorithms] and [can then do more than they did at the beginning].”

2. Are you aware of applications of artificial intelligence in anaesthesia?

English: “I don’t know exactly, but [I think many of these curve interpretations, [for example] of the pulse pressure curve, are based on something like that]. Also [ECG analysis always learns first before it spits something out].”

3. What do you think are the advantages and disadvantages of artificial intelligence in anaesthesia?

English: “Well, I would say that one advantage is that AI/ML [could objectify that gut feeling that you have from time to time]. For example, sometimes you think to yourself, regarding the duration of an operation or when you see certain parameters, ECG or EEG or something, “this could mean that”, but it can’t really be justified. And [I think artificial intelligence could provide better data there], so that you can place [that gut feeling] it on a firmer foundation. But, of course, [it’s error prone]. So, [if you’re not familiar with the procedure, how the data is created, then you can’t know what kind of errors can arise] and [you could rely too much on it]. For example, [if the measured values are subject to interference by other electronics, leading to some noise in the data or something like that].”

4. In particular, what do you think about the use of artificial intelligence to make predictions?
5. Which predictions do you think are most useful clinically?

English: “I haven’t given it much thought yet, but I think something that’s a bit better than what we have at the moment for [depth of anaesthesia] might be useful, because it only tells you that the patient is awake when you can already see it.”

Participant 18

1. What do you understand by the terms “artificial intelligence” or “machine learning”?

English: “That in this case, [computer programmes] are brought into the... to [collect data and to utilise it themselves] and [to become more intelligent], as it were, from [the collected data].”

2. Are you aware of applications of artificial intelligence in anaesthesia?

English: “Well, I don’t know for sure... so [there’s a lot of research going on right now, and we’ re also carrying out the ENVISION study using AI/ML], but [in everyday life I can’t think of anything like that].”

3. What do you think are the advantages and disadvantages of artificial intelligence in anaesthesia?

English: “Ultimately, [the system is only as good as the data it is trained on]. [In medicine, for example, there is often gender bias, especially in drug studies, and that is a challenge to overcome]. But [as an anaesthetist it is often important to take a step back and re-evaluate a situation and not blindly rely on the fact that the current course of action must be the right one].”

4. In particular, what do you think about the use of artificial intelligence to make predictions?
5. Which predictions do you think are most useful clinically?

English: “[I work mainly in anaesthesiology and not so much in intensive care, where you have an overview of patients over a long period of time or where it might make more sense to have the system evaluate a trend]. The [interaction of medicines] would be very good, because you often can’t keep track of that, and the system might look at [relaxation] or [anaesthetic depth measurement].”

Participant 19

1. What do you understand by the terms “artificial intelligence” or “machine learning”?

English: “[Software] that is [fed with lots of data] and is then [able to analyse these data itself] and then [can make a decision or give a recommendation]. For example, software that has “seen” hundreds of thousands of errors, can then in the future decide for itself: [“okay, in this case, what was the and the”? “What will be the outcome of a given therapy?”]”

2. Are you aware of applications of artificial intelligence in anaesthesia?

English: “No. So what I’ve heard is that [in intensive care it might be used at some point], and I’ve heard that [projects are going on with COVID patients].”

3. What do you think are the advantages and disadvantages of artificial intelligence in anaesthesia?

English: “[With respect to these two examples, predicting postoperative mortality and surgery duration, that can be time-consuming]. And with respect to [risk assessments and how to carry out an anaesthetic], I think a big disadvantage is, [if you decide to go against that recommendation, then there’s kind of an ethical and moral dilemma, right? What if the patient then dies? Then you must ask, “What could I have done better?” Should one always do the therapy recommended by artificial intelligence? So that’s kind of very difficult]. But predictions only, not recommendations, I think they’re good. [My question is how to cope with the recommendation of a particular therapy. If you make a different decision than the artificial intelligence and then something bad happens, then that’s a big question there]. But prediction, for example of mortality, I think that’s a good thing. Because [an AI has experience of many more cases than I do. And then the AI can say, yes, with this procedure and this patient, these complications are to be expected]. That’s fine. But [if it [the AI/ML] says, okay, for this operation on this patient, you must do this and that, and then for example a senior doctor decides they don’t want to do that, then the whole thing becomes a bit difficult].”

4. In particular, what do you think about the use of artificial intelligence to make predictions?

English: “[That it’s good]. [Systems that work with a lot of data, a lot of cases. They have a big advantage]. So, [I think that’s good].”

5. Which predictions do you think are most useful clinically?

English: “For example, on the intensive care unit, for example, during COVID. Maybe it would have been better if we had somehow, uh, well, [if we had somehow known which patients need ECMO, and which ones don’t?] Or [which patients need an escalation of treatment or something]? So, we could say, [this patient is so old, has these pre-existing conditions, and so the artificial intelligence says okay, from the like 1 million cases analysed, what percentage of this patient then ends up in intensive care units, what percentage on ECMO].... But then we know, okay, [with this patient we should pay a little bit more attention and start earlier with like NIV or something]. And for example, that’s good. That would be good.”

Participant 20

1. What do you understand by the terms “artificial intelligence” or “machine learning”?

English: “By artificial intelligence I understand that [various [parameters and values] that I normally interpret are taken over by computers], that [computers support me in interpreting parameters, and do not just calculate and present these parameters]… and can then support me accordingly in the decision-making process. And by machine learning I understand that the [algorithms behind this process] [improve their interpretation over time], and can perhaps also [make predictions].”

2. Are you aware of applications of artificial intelligence in anaesthesia?

English: “I know [in one of our intensive care units there is a warning signal that flashes when the glucose concentration is too low]. I wonder if you could call that artificial intelligence? That’s an extra pop-up that you must click away, so that you can continue working on the computer. Otherwise, [I at least haven’t worked with it].”

3. What do you think are the advantages and disadvantages of artificial intelligence in anaesthesia?

English: “So, an advantage, I think, [is it can support you in making decisions, especially when you are uncertain. If parameters are perhaps contradictory, it can support you]. And I think, even though I’m not really a technology freak, that [computers can probably do that better than humans. We rely a lot on experience, on feeling, and that is sometimes justified. But I think that in some areas, artificial intelligence is more precise and perhaps makes better decisions than humans]. The disadvantage is that [anaesthetists will perhaps become superfluous at some point if the AI can do that, do it better than we can]. That would be a pity, of course, but if that’s the case, then so be it. The second thing is that I fear that [competence may be lost if one only relies on these warning systems and no longer learns to interpret things oneself]. This is especially true for colleagues who learn and work with such systems from the very beginning and know nothing else. And in general, even if you don’t [lose competence], [you might still become a bit more careless and a bit lazy because you rely too much on this machine]. Yeah, that’s what I think.”

4. In particular, what do you think about the use of artificial intelligence to make predictions?

English: “[Totally ambivalent]. I think [on the one hand that it can be a really good, supporting tool and that it makes treatment in general safer, probably]. And as I said before, [I think that artificial intelligence can probably make smarter decisions than humans. Especially in stressful situations]. But I also see the disadvantages that I have just described, with the [possible loss of competence on the part of the anaesthetists] [and the loss of jobs. We might make ourselves totally superfluous at some point]. [That’s why I can’t really say in general. In general, I would say that I tend to have a positive attitude, but not 100%].”

5. Which predictions do you think are most useful clinically?

English: “[Everything that acutely endangers the patient]. For example, [hypoglycaemia]. Of course, as an anaesthetist you should notice that, but the computer can of course support you. And maybe there are [things that are quite subtle, and that could easily be overlooked]. Um. If a prediction says that [the body temperature], for example, will fall slowly. That’s not so urgent, but you might miss it if it’s so very slow. So, these two things, [the very acute, very dangerous things] and the [very insidious things that perhaps slip through your fingers].”

Participant 21

1. What do you understand by the terms “artificial intelligence” or “machine learning”?

English: “Okay, so I would say artificial intelligence is, in some sense, [decision-making by computer systems] themselves. Machine learning is [learning] [by means of computer programmes].”

2. Are you aware of applications of artificial intelligence in anaesthesia?

English: “In terms of feedback systems... in the case of feedback, it is a reflection, but decision-making... [no, not really].”

3. What do you think are the advantages and disadvantages of artificial intelligence in anaesthesia?

English: “[It can be used as a support tool for decision-making]. The disadvantage, of course, as with all predefined things, is that [there is also the danger that you no longer make your own decisions, but only rely on what the [artificial] intelligence tells you to do]. So, [it’s based on blind trust].”

4. In particular, what do you think about the use of artificial intelligence to make predictions?

English: “[It’s definitely very exciting]. [I can’t quite imagine how that would work]. But [presumably if enough data is mined, it’s certainly something that could be used to help avoid bad decisions].”

5. Which predictions do you think are most useful clinically?

English: “[Making predictions that could be critical for patients], [for example, when you enter dosages, so that you don’t make a gross mistake in, for example, paediatric anaesthesia]. Or also [things that you simply don’t pay attention to], for example [temperature], or similar things that can cause a lot of harm to patients.”
